# Speed-up hyperspheres homotopic path tracking algorithm for PWL circuits simulations

**DOI:** 10.1186/s40064-016-2534-5

**Published:** 2016-06-24

**Authors:** A. Ramirez-Pinero, H. Vazquez-Leal, V. M. Jimenez-Fernandez, H. M. Sedighi, M. M. Rashidi, U. Filobello-Nino, R. Castaneda-Sheissa, J. Huerta-Chua, L. A. Sarmiento-Reyes, J. R. Laguna-Camacho, F. Castro-Gonzalez

**Affiliations:** Facultad de Instrumentación Electrónica, Universidad Veracruzana, Cto. Gonzalo Aguirre Beltrán S/N, 91000 Xalapa, Veracruz Mexico; Department of Mechanical Engneering, Shahid Chamran University, Ahvaz, Iran; Shanghai Key Lab of Vehicle Aerodynamics and Vehicle Thermal Management Systems, Tongji University, 4800 Cao An Rd., Jiading, Shanghai, 201804 People’s Republic of China; ENN-Tongji Clean Energy Institute of Advanced Studies, Shanghai, People’s Republic of China; Facultad de Electrónica y Comunicaciones, Universidad Veracruzana, Venustiano Carranza S/N, 93390 Poza Rica, Veracruz Mexico; Facultad de Ingeniería Mecánica Eléctrica, Universidad Veracruzana, Venustiano Carranza S/N, 93390 Poza Rica, Veracruz Mexico; Instituto Nacional de Astrofísica, Óptica y Electrónica, Luis Enrique Erro #1, 72000 Sta. María Tonantzintla, Puebla Mexico

**Keywords:** Multiple operating points, Homotopy continuation method, Piecewise linear, Path tracking algorithm, Nonlinear circuits

## Abstract

In the present work, we introduce an improved version of the hyperspheres path tracking method adapted for piecewise linear (PWL) circuits. This enhanced version takes advantage of the PWL characteristics from the homotopic curve, achieving faster path tracking and improving the performance of the homotopy continuation method (HCM). Faster computing time allows the study of complex circuits with higher complexity; the proposed method also decrease, significantly, the probability of having a diverging problem when using the Newton–Raphson method because it is applied just twice per linear region on the homotopic path. Equilibrium equations of the studied circuits are obtained applying the modified nodal analysis; this method allows to propose an algorithm for nonlinear circuit analysis. Besides, a starting point criteria is proposed to obtain better performance of the HCM and a technique for avoiding the reversion phenomenon is also proposed. To prove the efficiency of the path tracking method, several cases study with bipolar (BJT) and CMOS transistors are provided. Simulation results show that the proposed approach can be up to twelve times faster than the original path tracking method and also helps to avoid several reversion cases that appears when original hyperspheres path tracking scheme was employed.

## Background

Circuit simulation is an important phase during the development of new electronic circuits. As for the integrated circuit design, this phase is vital and requires advanced tools capable to perform faster and accurate analysis. The DC analysis, also known as operating point calculation, commonly is the first step in circuit analysis. For a nonlinear circuit, the DC analysis provides a nonlinear algebraic equation system (NAES). The NAES, usually, is solved using the Newton–Raphson Method (NRM). Nevertheless, this method occasionally fails, leading to oscillations or diverging to infinity; another shortcoming of the method is its inefficiency to find multiple operating points. The NRM method performs in such a way that once a solution is found, stops and no further operations are performed. As an alternative, the homotopy continuation method (HCM) (Jimenez-Islas et al. 2013; Oliveros-Munoz and Jimenez-Islas 2013; Jimenez-Islas 1996; Bates et al. 2008; Ushida et al. 2002; Melville et al. 1993; Vazquez-Leal et al. 2005, 2011a, b, 2012, 2013; Yamamura et al. 1999; Kuno and Seader 1988; Watson 1986, 2009; Sosonkina et al. 1996; Watson et al. 1987; Allgower and Georg 1993, 1997; Gritton et al. 2001; Trajkovic et al. 1990; Verschelde 2011; Gunji et al. 2003; Torres-Munoz et al. 2014) was developed to find multiple solutions providing good convergence characteristics (Watson 1990). Besides, HCM has been applied to calculate multiple operating points of circuits containing components described by exponential (Vazquez-Leal et al. 2011b), polynomial (Torres-Munoz et al. 2014), or PWL (Vazquez-Leal et al. 2014) models.

Recently, analysis based on piecewise-linear modeling has emerged and gained popularity in circuit simulation and other related areas (Vazquez-Leal 2013; Guerra-Gómez et al. 2013; Junaid and Wang 2006; D’Arco and Suul 2014; Li et al. 1997; Lin and Wang 2009). This kind of analysis is based on replacing traditional nonlinear models by piecewise-linear (PWL) approximations (Trejo-Guerra et al. 2013, 2012; Jimenez-Fernandez et al. 2013a, b). This approach helps to reduce the complexity of equations, which practically are linear. Unfortunately, it implies a trade-off because the number of subdivided linear regions that must be computed to obtain an acceptable solution accuracy. This strategy helps to reduce convergence issues that may arise when performing numerical analysis (Roos and Valtonen 1999), although it has to deal with the task of providing an adequate description of the nonlinear device (Jimenez-Fernandez et al. 2007). To achieve this, several methodologies have been proposed to find multiple solutions of PWL circuits (Pastore 2009; Yamamura and Yomogita 2000; Ying et al. 2008; Yamamura and Ohshimar 1998; Yamamura 1993a; Yamamura and Tanaka 2000; Tadeusiewicz and Halgas 1999; Pastore and Premoli 1993; Katzenelson 1965; Yammamura and Horiuchi 1990; Stevens and Lin 1981; Eyndhoven 1986; Tadeusiewicz and Kuczynski 2013). Nevertheless, these methodologies exhibit some drawbacks like the requirement of several simulations to find multiple solutions (Katzenelson 1965; Yammamura and Horiuchi 1990), the use of implicit PWL models (Stevens and Lin 1981; Eyndhoven 1986), or the need to provide circuit equations in terms of the linear complementary problem (LCP), which implies to compute state variable models (Tadeusiewicz and Kuczynski 2013). The use of implicit models means that the number of linear regions may become useless when node synthesis is applied. Besides, compared to explicit models, implicit PWL models require a more complex algorithm to compute the model state variables.

Among all piecewise-linear models, the canonical piecewise-linear model proposed by Chua has been widely accepted due to its compact structure (De Jesus-Ventura et al. 2009). This model describes a device in a compact global representation, taking into account its two terminal V–I characteristic. This model describes a device in a concise representation, taking into account its two terminal V–I characteristics. Because no redundant data is stored, this approach greatly reduces the memory space required for storing device parameters. Besides, due to the continuity of this model, it is no required to store information about the boundaries for each linear region. All these characteristics makes Chua’s canonical PWL model a suitable option to be employed in circuit analysis. In Vazquez-Leal et al. (2014) it was proposed for the first time a numerical continuation technique of homotopy trajectories for PWL circuits that is based on path tracking of hyperspheres centered over the homotopy curve. The advantages of this technique are: uses Chua’s canonical PWL model and does not require to express equations in terms of the LCP. Therefore, this work will use the path tracking method proposed in Vazquez-Leal et al. (2014) and will perform some modifications to reduce computing time without losing accuracy. Also, we propose a method capable to avoid the reversion phenomenon (Yamamura 1993b). Additionally, a selection criteria for the path tracking starting point is addressed.

This paper is organized as follows. “Original HCM scheme for studying PWL circuits” section provides a brief description on PWL modelling, a short introduction to the HCM method, and a summary of the proposed method in Vazquez-Leal et al. (2014). The suggested technique for avoiding the reversion phenomenon, the proposed path tracking method, and selection criteria for the starting point are provided in “Proposed homotopy scheme” section. In “Cases study” section, five cases study of nonlinear circuits are presented and solved using an HCM and the proposed path tracking method. Numerical simulations and discussion about results are provided in “Numerical simulation and discussion” section. Finally, our conclusions about this work are given in “Conclusions” section.

## Original HCM scheme for studying PWL circuits

### Equilibrium system of equations

The equilibrium system of equations are obtained applying the modified nodal analysis (MNA) (Ho et al. 1975), which is a method that allows the systematic study of circuits containing devices incompatible with the classic nodal analysis like voltage sources, voltage-dependent voltage sources, among others.

As a result of the MNA a set of equations of the form

1 $$\begin{aligned} {\mathbf {f}}({\mathbf {x}})={\mathbf {0}} \end{aligned}$$

will be obtained; where $\mathbf {x}$ represents the vector of unknowns (electrical variables) of the circuit.

### Piecewise linear (PWL) model

The Piecewise Linear Model is an approximation of a nonlinear equation to a set of linear equations which, altogether, exhibit the same behavior as the original system. In this work, Chua’s model serves as base for the proposed homotopy scheme; this model is described as follows

2 $$\begin{aligned} y(x)=a+bx+ {\sum _{i=1}^{\sigma } c_i|x-\beta _i|}, \end{aligned}$$

model parameters are computed by

3 $$\begin{aligned} a& = {} y(0)- {\sum _{i=1}^{\sigma } c_i|\beta _i|}, \quad b& = {} \frac{J_1+J_{\sigma +1}}{2}, \quad c_i& = {} \frac{J_{i+1}-J_{i}}{2}, \quad i=1,2,\ldots \sigma , \end{aligned}$$

where $\beta$ represents the breakpoints, $\Sigma$ represents the number of breakpoints, and $J_i$ represents the slope of the *i*-th straight line segment in the PWL model.

### Homotopy continuation method (HCM)

To solve a system of equations using the HCM, first, the actual solution is introduced in a set of solutions described by

4 $$\begin{aligned} {\mathbf {H}}({\mathbf {f}}({\mathbf {x}}),\lambda )={\mathbf {0}}, \quad {\mathbf {H}} \in {\mathfrak {R}}^n \times {\mathfrak {R}} \rightarrow {\mathfrak {R}}^n, \end{aligned}$$

where $\lambda$ is the homotopic parameter and $\mathbf {f(x)}$ is the system of equations to be solved. To find the solution of the original system, the HCM starts from a known solution of the homotopic system, which is commonly given at $\lambda =0$. Afterwards, a path tracking method is employed to calculate subsequent points within the homotopic curve. Each time the homotopic path intercepts the solution line (generally placed at $\lambda =1$) a solution to the original system is found (Vazquez-Leal et al. 2011a).

The HCM is commonly used to find multiple solutions as it does not stop the calculation of solutions once it has found one, unlike the NRM which is designed to find just one solution per simulation.

An important issue of the HCM is the possibility that path tracking fails by following an incorrect path, this will cause losing solutions or not finding any solution at all, even if the Homotopy path exist and several, or all, solutions may be located.

### Homotopy formulation

#### Homotopy formulation

The homotopy formulation used in this work is Newton’s homotopy given by

5 $$\begin{aligned} {\mathbf {H}}({\mathbf {f}}({\mathbf {x}}),\lambda ) = {\mathbf {f}}({\mathbf {x}}) + (\lambda -1) {\mathbf {f}}({\mathbf {x}}_i) = {\mathbf {0}}. \end{aligned}$$

As reported in Vazquez-Leal et al. (2014), it is possible to model devices using PWL techniques by applying Newton’s Homotopy during DC analysis. Nevertheless, it is important to notice that experiments in Vazquez-Leal et al. (2014) proved that homotopic curves are also PWL as long as Newton’s Homotopy is employed. This important characteristic will be applied in this work to propose a novel initial point selection scheme and a new scheme to accelerate the trace for the homotopic path.

#### Modified spheres algorithm (MSA)

In Vazquez-Leal et al. (2014) the modified spheres algorithm path tracking method was used to find the homotopic curve. This method consists in including the equation of a sphere within the original homotopic system (Vazquez-Leal et al. 2011b; Torres-Munoz et al. 2014; Yamamura 1993b; Oliveros-Munoz and Jimenez-Islas 2013; Jimenez-Islas 1996). It is expressed as

6 $$\begin{aligned} \begin{aligned} S(x_{1},x_{2},\dots ,x_{n},\lambda )&=(x_{1}-c_{1})^{2} +(x_{2}-c_{2})^{2} +\cdots +(x_{n}-c_{n})^{2}\\&\quad +(\lambda -c_{n+1})^{2}-r^{2}=0, \end{aligned} \end{aligned}$$

where *c* is the center of the sphere, *r* is its radius, and *n* is the number of variables from the equilibrium system of equations. By incorporating (6) into the homotopic system (4) the system of equations become

7 $$\begin{aligned} H_{1}(f_{1}(x),\lambda )&=0, \\ H_{2}(f_{2}(x),\lambda )&=0, \\&\vdots \\ H_{n}(f_{n}(x),\lambda )&=0, \\ S(x_{1},x_{2},\cdots ,x_{n},\lambda )&=0, \end{aligned}$$

the system contains $n+1$ equations and $n+1$ variables.

**Fig. 1:**
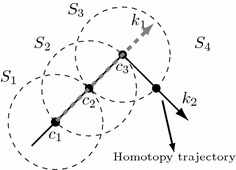
PWL homotopy path tracking using the MSA (Torres-Munoz et al. 2014)

Figure 1 shows the application of the MSA. When the center of the sphere is located at ${\mathbf {c_1}}$, the NRM is applied with predictor vector ${\mathbf {k_1}}$; corrector steps are applied to achieve the intersection between the sphere and the homotopic path which will be used as center of the next sphere $S_2$.

## Proposed homotopy scheme

### Starting point criteria

Chua’s model possess the characteristic of having upper and lower limits, this means that its linear behavior will not suffer any change because there are no breakpoints affecting its linearity. In addition, when using an HCM, the starting point is selected far from the interest area as a means to induce the homotopy to run through an entire region where a solution may be located, this is known as feasible region. Therefore, two tests are proposed for the selection of the starting point.

**Fig. 2:**
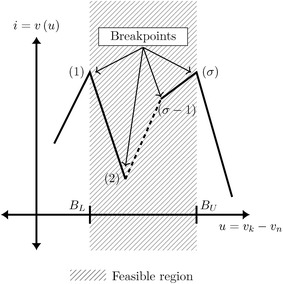
Starting point criteria

- *Test 1* Every value of the starting point vector must not have a value located within the feasible region. In electrical terms, the values for voltage variables must be higher (or equal) than the highest positive voltage supply or lower (or equal) than the lowest negative voltage supply; the values for current variables should be in a higher range than the possible normal operating currents for the circuit, for instance, flip-flops typically work in the range of milliamperes so setting the values in the range of amperes would be suitable for current variables.
- *Test 2* This work proposes circuits modeled by devices of type $i=y(u)$. Therefore, the test will focus on this kind of elements. Nevertheless, a simple extrapolation of the explanation in this section may be extended to elements of type $u=y(i)$. As it has been explained in Test 1, it is important that initial point should be located above or below the maximum and minimum values of the power supply, respectively. By doing this, the chance of the homotopic path to cross all the feasible region of solutions is increased. However, it is important to understand that initial point consist in a set of electrical values (nodal currents and nodal voltages). Therefore, given the nature of the MNA formulation, most of the electrical variables for the circuits under study are nodal voltages, being the only variable of type current the unknown current from the power source. Besides, we know that every PWL device has an specific number of breakpoints ($\Sigma$) and within them are breakpoints that may cause multiple operating points. Therefore, it is important to assure that proposed initial point in terms of nodal voltages ($v_k$ and $v_n$ for each pair of terminals of the PWL devices), produce a voltage drop *u* outside the bounded region by the lower breakpoint ($B_L$) and upper breakpoint ($B_U$) as it can be seen in Fig. 2. The shaded region is the feasible region for solutions although solutions, in fact, could be located out of this region for some devices. Therefore, Test 1 and Test 2 are complementary; helping to propose initial points capable to provide the most number of solutions for simulation or path tracking.

### Avoiding the reversion phenomenon

The MSA path tracking method consists in calculating the point where the circumference of a sphere crosses the homotopic path; nevertheless, this sphere always intersects the homotopic path in two points, as for our purposes we are only interested in just one. It is possible that the NRM calculations for the new point in the path converge to a point already found, this situation cause a backward path tracking, thus causing the method to fail. This situation is known as the reversion phenomenon (Yamamura 1993b).

This work introduces a technique capable to avoid the reversion phenomenon. It consists in perturbing the hypersphere equation (6) to avoid one of the interceptions between the hypersphere and the homotopic path.

**Fig. 3:**
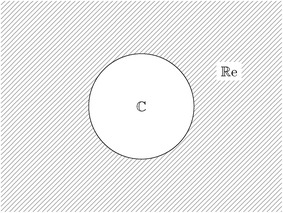
Graphical inverted sphere domain

To understand the proposed technique, it is necessary to study the concept of an inverted sphere which is described by

8 $$\begin{aligned} S_{inv}(x_{1},x_{2},\ldots ,x_{n+1})&= \bigl ((x_{1}-c_{inv_1})^{2}+(x_{2}-c_{inv_2})^{2}+\cdots \bigr . \\&\bigl . \qquad +(x_{n+1}-c_{inv_{n+1}})^{2}-r_{inv}^{2} \bigr ) ^ {1/2}\ge 0, \end{aligned}$$

where $x_{n+1}$ represents the homotopic variable ($\lambda$), $c_{inv}$ represents the inverted sphere center and $r_{inv}$ represents its radius. This equation is the same as obtaining the square root of (6). When substituting any value of ${\mathbf {x}}$ located inside the sphere in (8), it will generate a negative number inside the square root and create an empty region in the domain of real numbers as shown in Fig. 3.

In order to take advantage of the properties from the inverted sphere we add (6) and (8), placing the center of the inverted sphere at the unwanted interception and the center of the original sphere at the point obtained in the previous iteration, the result is

9 $$\begin{aligned} S_{new}(x_{1},x_{2},\ldots ,x_{n+1})&= \bigl ( 1/K_{inv} \bigr ) \bigl ( (x_{1}-x_{j-1,1})^{2}+(x_{2}-x_{j-1,2})^{2}+\cdots \bigr . \\\bigl . & \qquad +\,(x_{n+1}-x_{j-1,n+1})^{2}-r_{inv}^{2} \bigr ) ^ {1/2} +(x_{1}-x_{j,1})^{2} \\& \qquad +\,(x_{2}-x_{j,2})^{2} +\cdots +(x_{n+1}-x_{j,n+1})^{2} \\&\qquad -r^{2}=0, \end{aligned}$$

here *j* represents the *j*-th iteration and $K_{inv}$ is an arbitrary constant used to reduce the contribution of the inverted sphere to the equation in order to deform the shape of the sphere as little as possible.

**Fig. 4:**
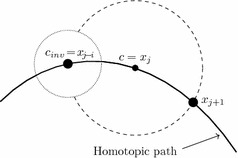
MSA with an inverted hypersphere

As shown in Fig. 4, the resulting system has a cavity in its circumference. In fact, this empty spot covers the unwanted interception of the original sphere and the homotopic path. This factor allows the NRM to find the next point in the homotopic path as it is the only solution for

10 $$\begin{aligned} H_{1}(f_{1}(x),\lambda )&=0, \\ H_{2}(f_{2}(x),\lambda )&=0, \\&\vdots \\ H_{n}(f_{n}(x),\lambda )&=0, \\ S_{new}(x_{1},x_{2},\ldots ,x_{n},\lambda )&=0, \end{aligned}$$

which is the new system of equations to trace the homotopic path.

**Fig. 5:**
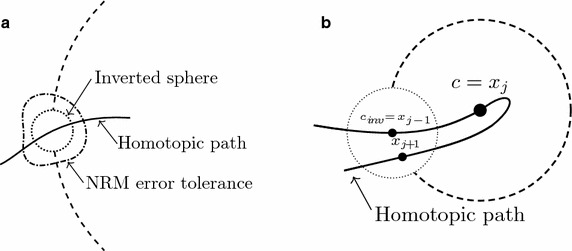
Inverted sphere problems in the MSA. **a** Small radius problem. **b** Big radius problem

In order to avoid a possible oscillation of the NRM or iterations with complex numbers, when it approaches the empty region, a limit in the number of iterations is established. When the limit is reached, the NRM is executed with a different starting point as described in Torres-Munoz et al. (2014). Empirically, we found that the radius of the inverted sphere should be smaller than the radius of the original one, a range between 1,000 to 10,000 times smaller is proposed in this work. If the inverted sphere radius is too small, the NRM might find the unwanted root even if it does not exist, because the radius from the inverted sphere is smaller than the error tolerance from the NRM as seen in Fig. 5a. If it is too big, the homotopic path tracking may fail because next point in the path could be inside the empty region of the original sphere as shown in Fig. 5b.

### Speed-up hyperspheres path tracking method (SHPT)

**Fig. 6:**
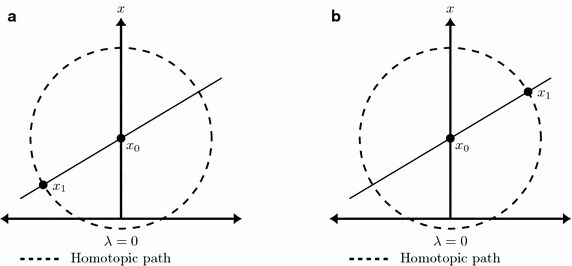
First iteration of the path tracking. **a** Case with negative solution found. **b** Case with positive solution found

**Fig. 7:**
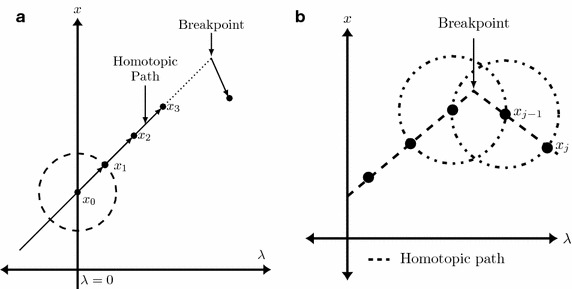
Path tracking method. **a** Straight line segment iterations. **b** PT method in breakpoints

This work proposes a modification to the path tracking method presented in Vazquez-Leal et al. (2014). It is capable to reduce the computing time for tracking homotopic paths having PWL characteristics. Taking advantage of the local linearity of PWL models, a parameterized straight line equation is deduced from the first two points ($x_0$ and $x_1$) obtained using the MSA (see Fig. 6). After that, this linear equation is used to calculate the next point ($x_2$) in the straight line and the values obtained are substituted in the homotopic system of equations, if these values satisfy the system of equations, the next iteration will be performed in the same way to find ($x_3, x_4,\ldots$) as depicted in Fig. 7a; otherwise, it means that we found a break point and the MSA must be used again two times in order to obtain a new straight line equation as depicted in Fig. 7b.

#### Homotopy formulation

The Newton Homotopy should be formulated based on (1). As shown in Vazquez-Leal et al. (2014), the homotopic curves obtained with this formulation have a PWL nature, this will hold as long as all of the nonlinear devices in the circuit are PWL modelled. This property allows to execute the following steps for the proposed path tracking method.

#### Starting point criteria

Once the homotopic system is defined, the SP should be arranged in a way to accomplish both Test 1 and Test 2 (see Fig. 2).

#### Modified hypersphere equation (hypersphere iterations)

Once the starting point (${{\mathbf {x}}}_{{\mathbf {0}}}$) is defined, the sphere equation (6) should be formulated with a center located at ${\mathbf {c}}={{\mathbf {x}}}_{{\mathbf {0}}}$. With this formulation the NRM must be applied to (10). If the found solution is within the region of $\lambda < 0$ (Fig. 6a), it will be used as the center of the inverted sphere for the next iteration and the center of the normal sphere will be located at ${{\mathbf {x}}}_{{\mathbf {0}}}$ in order to induce the NRM to find the interception on the positive side of $\lambda$ and follow the path heading to $\lambda =1$. If the found solution is within the region of $\lambda >0$ (Fig. 6b), the next sphere will have a center located at ${{\mathbf {x}}}_{\mathbf {1}}$ and the inverted sphere will have a center at ${\mathbf {x}}_{\mathbf {0}}$.

#### Straight line equation formulation

Once the direction of the path tracking has been set to the positive region of $\lambda$, it is possible to formulate the straight line equation given by

11 $$\begin{aligned} {\mathbf {x_{j+1}}}&={\mathbf {m}\lambda _{j+1}}+{\mathbf {x_{j-1}}}-{\mathbf {m}}\lambda _{j-1}, \\ {\mathbf {m}}&=\frac{{\mathbf {x}}_{\mathbf {j}}-{{\mathbf {x}}}_{\mathbf {j}-{\mathbf {1}}}}{ \lambda _{j}-\lambda _{j-1}}, \end{aligned}$$

where *j* represents the *j*-th iteration and *m* represents the slope of the straight line that crosses from $\mathbf {x_j}$ to ${{\mathbf {x}}}_{{\mathbf {j}}-{\mathbf {1}}}$.

Next iterations will be predicted substituting $\lambda _{j+1}=2\lambda _{j}-\lambda _{j-1}$ in (11), as shown in Fig. 7. If the values calculated using (11) satisfy (7) it can be assured that they belong to the homotopic path. This procedure shall be repeated for next predictions until the obtained values no longer satisfies (7). It means that iterations with straight lines found a breakpoint on the homotopic path just like the one in Fig. 7b. Therefore, two new iterations using the hypersphere tracing point should be performed to create a new straight line and predict points for the new segment.

The straight line path tracking does not need any correcting steps like those needed when using the NRM, this results in greatly reducing the computing resources and time required. It also avoids the calculation of different starting points when NRM fails. Furthermore, the diverging issues present sometimes in the NRM are moderated for these iterations. Finally, no reversion phenomenon will appear as (11) has only one solution and it leads to the forward path tracking.

#### Path tracking technique near breakpoints

When the homotopic path crosses a breakpoint of the PWL model (see Fig. 7b), (11) will not satisfy (7). At this point, (10) has to be solved, placing the center of the noninverted sphere at the last calculated point that was part of the homotopic path and the center of the inverted sphere at the second to the last calculated point; NRM is applied to calculate the next point in the path. Afterwards, another point should be calculated by solving (10) in order to repeat the hypersphere iterations procedure explained in “Straight line equation formulation” section. When a path has a high density of straight line segments, the SHPT will tend to slow down, although not as slow like the method proposed in Vazquez-Leal et al. (2014). This characteristic shows that SHPT, compared to MSA, requires lower computation time (CP) or could perform almost identical if the homotopic curve exhibits a high number of break points.

#### Find zero strategy

**Fig. 8:**
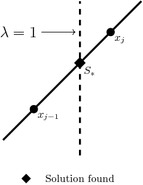
Find zero strategy

As reported in Vazquez-Leal et al. (2014), if the homotopic path crosses the solution line ($\lambda =1$), the exact solution to the PWL system can be obtained by calculating (11) using the last point before the homotopic path crossed the solution line and the next point after it, as shown in Fig. 8, and substituting $\lambda _{j+1}=1$. In other words, a linear interpolation at $\lambda =1$ is performed among the two iterations crossing $\lambda =1$.

### SHPT algorithm

In this section we introduce an algorithm for the SHPT method.

**Fig. 9:**
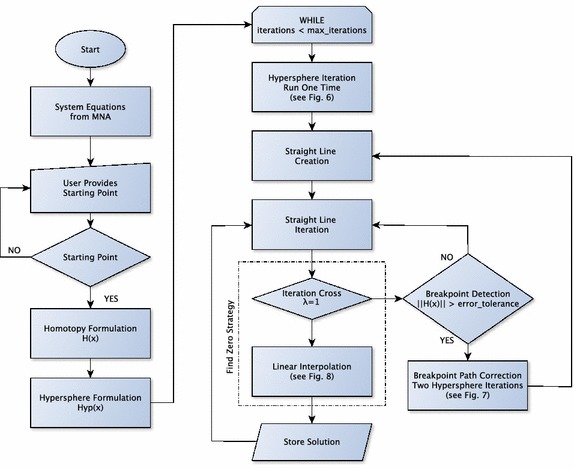
Flow diagram for the SHPT algorithm

The general procedure work as follows: first, the system of equations is generated as shown in (14). Second, the user provides a starting point. This starting point has to fulfill Test 1 and Test 2 already introduced in the previous section. For the case when starting point does not fulfill any of the tests, the user is requested to provide another starting point and the verification process is performed until a valid point is achieved. Then, the process continues by generating the Homotopic formulation for the system. Afterwards, the hypersphere formulation is given. It is important to mention that, at this point, the inverse hypersphere formulation is also generated to avoid the reversion phenomenon. Notice that the hypersphere and inverse hypersphere formulation will be updated after every iteration by adjusting its center. Once all necessary equations are already provided and a valid starting point is given, the main loop is started. It begins with a hypersphere iteration, the result of the iteration and the starting point allows the calculation of another point that allows calculation of a predictive straight line in the next block. Once this step is done, the Straight Line Iteration block is performed as explained in the corresponding section. As iteration continues, detection for crossing at $\lambda =1$ is performed; for the case that it is not detected, break point detection is applied. If a break point is not found, the process returns to the Straight Line Iteration block. For the case that a break point is detected, two hypersphere iterations are performed to correct the homotopic path (see Fig. 7). When $\lambda =1$ is located, linear interpolation (see Fig. 8) is applied to provide a very accurate approximation that is stored in a solution vector. Once the store block is executed, the process returns to the straight line iteration block. The algorithm will repeat until maximum number of iterations limit is reached. Figure 9 shows the flow diagram for the entire process.

## Cases study

In this section we provide five circuits to be analysed using the proposed path tracking method. All the proposed SP for the circuits fulfils the Test 1 and Test 2 starting point criteria. Also, we compare the proposed path tacking method against the proposed in Vazquez-Leal et al. (2014), this comparison includes the results using the inverted sphere and without it. The value for $K_{inv}$ was set to 50000 for all the cases.

### Example 1

**Fig. 10:**
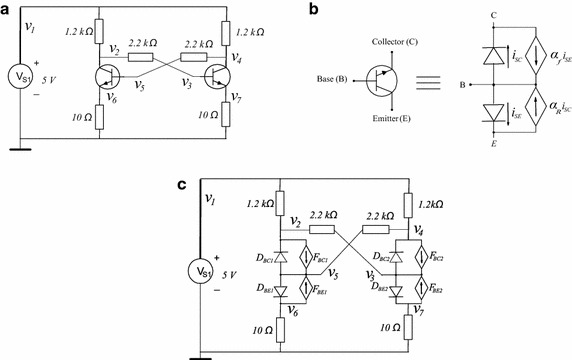
Example 1: Schematic Circuits. **a** Schematic Circuit for example 1. **b** Ebers–Moll model. **c** Schematic Circuit for example 1 with the Ebers–Moll model

The circuit in Fig. 10a (Tadeusiewicz and Kuczynski 2013) has two BJT Transistors which were modelled using the Ebers–Moll model shown in Fig. 10b. The PWL equations for these devices are

12 $$\begin{aligned} i_{BE}(v_B,v_E)&=0.1861|v_B-v_E-0.68|+1.4760|v_B-v_E-0.75| \\&\quad +9.8375|v_B-v_E-0.8|+27.7312|v_B-v_E-0.85| \\&\quad +67.1356|v_B-v_E-0.87|+106.3693|v_B-v_E| \\&\quad -91.0832, \\ i_{BC}(v_B,v_C)&=0.3685|v_B-v_C-0.68|+2.2023|v_B-v_C-0.75| \\&\quad +12.8961|v_B-v_C-0.79|+50.5505|v_B-v_C-0.84| \\&\quad +200.5718|v_B-v_C-0.87|+266.5949|v_B-v_C| \\&\quad -229.0503. \end{aligned}$$

Figure 10c shows the equivalent circuit once the Ebers–Moll has been substituted.

Applying the MNA method to the circuit, the equilibrium system of equations are obtained as

13 $$\begin{aligned} f_{1}&=0.16667\times 10^{-2}v_{1}-0.83333\times 10^{-3}v_{2}-0.83333\times 10^{-3}v_{4}+i_{V_{S1}}=0, \\ f_{2}&=-0.83333\times 10^{-3}v_{1}+0.12878\times 10^{-2}v_{2}-0.45454\times 10^{-3}v_{3} \\&\quad -i_{BC}(v_{5},v_{2})+0.99i_{BE}(v_{5},v_{6})=0, \\ f_{3}&=-0.45454\times 10^{-3}v_{2}+0.45454\times 10^{-3}v_{3}+0.5i_{BC}(v_{3},v_{4}) \\&\quad +0.01i_{BE}(v_{3},v_{7})=0, \\ f_{4}&=-0.83333\times 10^{-3}v_{1}+.12878\times 10^{-2}v_{4}-.45454\times 10^{-3}v_{5}-i_{BC}(v_{3},v_{4}) \\&\quad +.99i_{BE}(v_{3},v_{7})=0, \\ f_{5}&=-0.45454\times 10^{-3}v_{4}+.45454\times 10^{-3}v_{5}+0.5i_{BC}(v_{5},v_{2}) \\&\quad +0.01i_{BE}(v_{5},v_{6})=0, \\ f_{6}&=(1/10) v_{6}-i_{BE}(v_{5},v_{6})+0.5i_{BC}(v_{5},v_{2})=0, \\ f_{7}&=(1/10)v_{7}-i_{BE}(v_{3},v_{7})+0.5i_{BC}(v_{3},v_{4})=0, \\ f_{8}&=v_{1}-5=0, \end{aligned}$$

which is used as the base to create a Newton homotopy, and results in the following homotopic system of equations

14 $$\begin{aligned} H_{1}&=\lambda \bigl [ 0.16667\times 10^{-2}v_{1}-0.83333\times 10^{-3}v_{2}-0.83333\times 10^{-3}v_{4} \bigr . \\&\quad +i_{V_{S1}} \bigr ] (1-\lambda )(6.00166)=0, \\ H_{2}&=\lambda \bigl [ -0.83333\times 10^{-3}v_{1}+0.12878\times 10^{-2}v_{2}-0.45454\times 10^{-3}v_{3} \bigr . \\&\quad - i_{BC}(v_{5},v_{2}) +0.99i_{BE}(v_{5},v_{6}) \bigr ]+(1-\lambda )(-53.05737)=0, \\ H_{3}&=\lambda \bigl [-0.45454\times 10^{-3}v_{2}+0.45454\times 10^{-3}v_{3}+0.5i_{BC}(v_{3},v_{4}) \bigr . \\&\quad +0.01i_{BE}(v_{3},v_{7}) \bigr ]+(1-\lambda )(37.77025)=0, \\ H_{4}&=\lambda \bigl [ -0.83333\times 10^{-3}v_{1}+.12878\times 10^{-2}v_{4}-.45454\times 10^{-3}v_{5} \bigr . \\&\quad - i_{BC}(v_{3},v_{4})+.99i_{BE}(v_{3},v_{7}) \bigr ]+(1-\lambda )(-53.05737)=0, \\ H_{5}&=\lambda \bigl [ -0.45454\times 10^{-3}v_{4}+.45454\times 10^{-3}v_{5}+0.5i_{BC}(v_{5},v_{2}) \bigr . \\&\quad + 0.01i_{BE}(v_{5},v_{6}) \bigr ]+(1-\lambda )(37.76480)=0, \\ H_{6}&=\lambda \bigl [ (1/10)v_{6}-i_{BE}(v_{5},v_{6})+0.5i_{BC}(v_{5},v_{2}) \bigr ]+(1-\lambda )(15.89174)=0, \\ H_{7}&=\lambda \bigl [(1/10)v_{7}-i_{BE}(v_{3},v_{7})+0.5i_{BC}(v_{3},v_{4}) \bigr ]+(1-\lambda )(15.89174)=0, \\ H_{8}&=\lambda \bigl [ v_{1}-5 \bigr ]+(1-\lambda )(2)=0. \end{aligned}$$

The proposed starting point for the homotopic path tracking is shown in Table 1. Table 2 shows that the SP fulfils both Test 1 and Test 2 criteria. The first column contains the variables of the NAES and the PWL model dependent variables $u=v_k-v_n$, where *k* and *n* represent the diodes nodes; the second column shows that every variable is greater than or equal to the value of the voltage source ($V_{S1}=5$), accomplishing Test 1; the third column shows the fulfilment of Test 2 as every PWL model dependent variable $u=v_k-v_n$ is less than or equal to the lowest breakpoint ($B_L=0$) in (12).

**Table 1:** Starting Point (SP) for Example 1

Variable	Starting point (SP)
$v_{1}$	7
$v_{2}$	7
$v_{3}$	6
$v_{4}$	7
$v_{5}$	6
$v_{6}$	7
$v_{7}$	6
$i_{V_{S1}}$	6

**Table 2:** Test 1 and Test 2 proof

Variable	Test 1
$v_{1}$	7 ≥ 5
$v_{2}$	7 ≥ 5
$v_{3}$	6 ≥ 5
$v_{4}$	7 ≥ 5
$v_{5}$	6 ≥ 5
$v_{6}$	7 ≥ 5
$v_{7}$	6 ≥ 5
$i_{V_{S1}}$	6 ≥ 5

Voltage drop in PWL devices	Test 2
$v_5-v_2$	$-1\le 0$
$v_5-v_6$	$-1\le 0$
$v_3-v_4$	$-1\le 0$
$v_3-v_7$	$0\le 0$

**Fig. 11:**
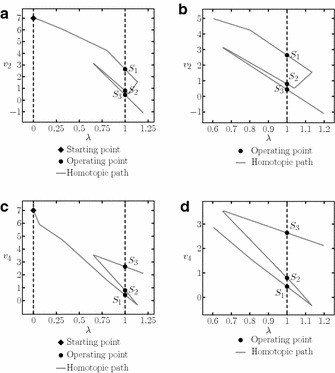
Homotopic paths from example 1. **a** Homotopic path for $v_{4}$. **b** Zoom to the homotopic path for $v_{4}$. **c** Homotopic path for $v_{2}$. **d** Zoom to the homotopic path for $v_{2}$

The resulting homotopic path when using the proposed SP is depicted in Fig. 11 for $v_4$ and $v_2$.

In Tadeusiewicz and Kuczynski (2013) three operating points were reported for this circuit, in Fig. 11 can be seen that all of them were found using the proposed methodology. The solutions found are listed in Table 3.

**Table 3:** Obtained operating points for example 1

Solution	$S_1$	$S_2$	$S_3$
Variable			
$v_{1}$	5	5	5
$v_{2}$	2.63687	0.79633	0.43999
$v_{3}$	0.73608	0.71936	0.41549
$v_{4}$	0.43999	0.79633	2.63687
$v_{5}$	0.41549	0.71936	0.73608
$v_{6}$	0.01116	0.03503	0.04652
$v_{7}$	0.04652	0.03503	0.01116
$i_{V_{S1}}$	$-$0.00576	$-$0.00700	$-$0.00576
Error$_{15}$	2.56330E−9	2.6000E−9	1.8497E−9
Error$_{7}$	7.65891E−9	5.5292E−9	1.3424E−8

The errors of the obtained solutions are calculated by

15 $$\begin{aligned} Error= \sqrt{\sum \limits _{i=1}^N {\mathbf {f}}({\mathbf {S}}_i)^2}, \end{aligned}$$

where *N* represents the number of equations in the original system and ${\mathbf {f}}({\mathbf {S}}_i)$ represents the substitution of the solution $S_i$ in the vector of equations (1).

### Example 2

**Fig. 12:**
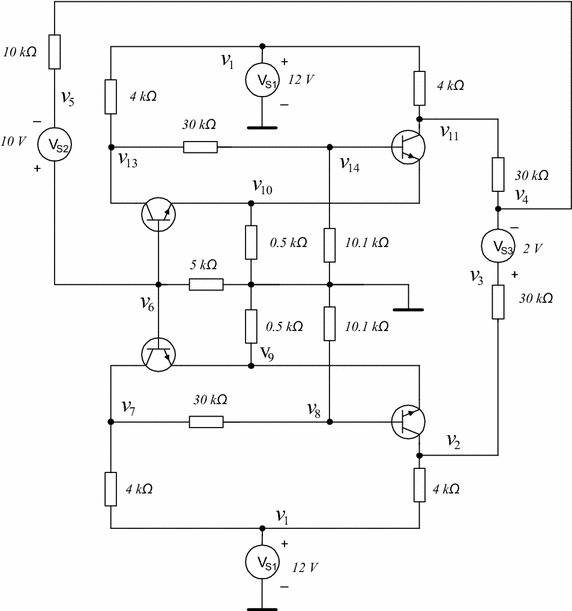
Nine solutions Chua’s circuit

**Fig. 13:**
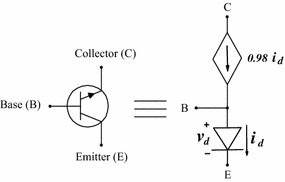
Simplified version of the Ebers–Moll model

The circuit shown in Fig. 12 is the classical Chua’s circuit, this circuit has nine solutions. The values of the resistors are taken from Reyes (1994) and BJT Transistors were modelled using a simplified version of the Ebers–Moll model, see Fig. 13. The V–I characteristics of the model in this model are given by

16 $$\begin{aligned} i_{D}(v_B,v_E)&=-0.05486 + 0.14827|v_B-v_E|+ 0.01157|v_B-v_E - 0.306| \\&\quad + 0.01181|v_B-v_E- 0.3375| + 0.04904|v_B-v_E - 0.366| \\&\quad + 0.07583|v_B-v_E - 0.3875|. \end{aligned}$$

The equilibrium system of equations obtained using the MNA has sixteen variables, thirteen are voltage variables and three are current variables from the three independent voltage sources.

In order to be able to calculate all the solutions of this circuit three SP were needed. These SPs are listed in Table 4 and all of them fulfil the starting point selection criteria proposed in this work.

**Table 4:** Starting points for example 2

Starting point	SP1	SP2	SP3
Variable			
$v_{1}$	−13	−14	−15
$v_{2}$	−13	−15	−15
$v_{3}$	−13	−16	13
$v_{4}$	−13	17	16
$v_{5}$	−13	18	−13
$v_{6}$	20	12	23
$v_{7}$	−13	16	−13
$v_{8}$	20	15	19
$v_{9}$	20	14	13
$v_{10}$	20	−14	21
$v_{11}$	−13	−16	−13
$v_{12}$	−13	−14	13
$v_{13}$	−13	−12	15
$i_{V_{S1}}$	−13	2	−1
$i_{V_{S2}}$	−13	2	−1
$i_{V_{S3}}$	−13	2	−1

**Fig. 14:**
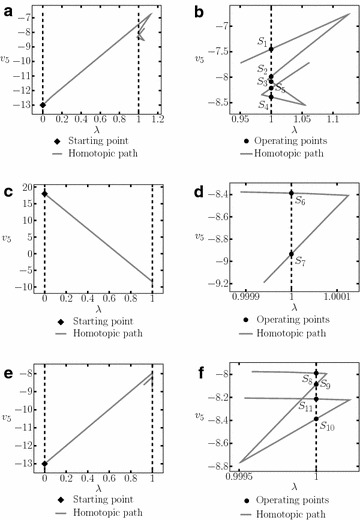
Homotopic paths for $v_5$ from Example 2. **a** Homotopic path for $v_5$ using SP1. **b** Zoom to **a**. **c** Homotopic path for $v_5$ using SP2. **d** Zoom to **c**. **e** Homotopic path for $v_5$ using SP3. **f** Zoom to **e**

The resulting homotopic paths for variable $v_5$ are shown in Fig. 14. A total of eleven solutions were found using three different SP, nevertheless, there are only nine different solutions because $S_5$ was found using the SP3 and SP2, and $S_9$ was found using SP1 and SP3.

Numerical solutions are listed in Tables 5 and 6.

**Table 5:** Operating points from Example 2 using SP1

Starting point	SP1				
Solution	$S_1$	$S_2$	$S_3$	$S_4$	$S_5$
Variable					
$v_{1}$	12	12	12	12	12
$v_{2}$	10.75096	10.43859	10.39498	4.84208	2.47682
$v_{3}$	1.38307	−1.27205	−1.64274	−2.96478	−2.38453
$v_{4}$	−0.61692	−3.27205	−3.64274	−4.96478	−4.38453
$v_{5}$	−7.45041	$-$7.98903	−8.08633	−8.38692	−8.21367
$v_{6}$	2.54958	2.01096	1.91366	1.61307	1.78632
$v_{7}$	−4.54315	−0.75565	−0.07147	7.38990	8.58443
$v_{8}$	−1.14428	−0.19032	−0.01800	1.62547	1.82001
$v_{9}$	2.16789	1.63661	1.54063	1.27021	1.45263
$v_{10}$	2.16789	1.69623	1.80832	1.80832	1.45263
$v_{11}$	10.51566	−0.83173	−2.34971	−2.50524	2.24153
$v_{12}$	−4.54315	10.13282	10.84530	10.84530	8.58443
$v_{13}$	−1.14428	2.07001	2.18505	2.18505	1.82001
$i_{V_{S1}}$	−0.00895	−0.00725	−0.00729	−0.00685	−0.00652
$i_{V_{S2}}$	−0.00068	−0.00047	−0.00044	−0.00034	−0.00038
$i_{V_{S3}}$	0.00031	0.00039	0.000401	0.00026	0.00016
$\hbox {Error}_{15}$	4.3457E−8	3.9249E−9	3.9283E−10	1.3055E−8	3.5880E−10
$\hbox {Error}_{7}$	4.0033E−7	1.2607E−7	1.1384E−7	1.0513E−7	1.6096E−7

**Table 6:** Operating points from Example 2, using SP2 and SP3

Starting point	SP2		SP3			
Solution	$S_6$	$S_7$	$S_8$	$S_9$	$S_{10}$	$S_{11}$
Variable						
$v_{1}$	12	12	12	12	12	12
$v_{2}$	−2.26995	−2.48607	−0.59643	−2.11441	−2.26995	2.47609
$v_{3}$	−2.96478	−4.80186	−1.27205	−1.64274	−2.96478	−2.38462
$v_{4}$	−4.96478	−6.80186	−3.27205	−3.64274	−4.96478	−4.38462
$v_{5}$	−8.38692	−8.93395	−7.98903	−8.08633	8.38692	−8.21370
$v_{6}$	1.61307	1.06604	2.01096	1.91366	1.61307	1.78629
$v_{7}$	10.84530	10.84531	10.13282	10.84530	10.84530	8.58478
$v_{8}$	2.18505	2.18505	2.07001	2.18505	2.18505	1.82007
$v_{9}$	1.80832	1.80832	1.69623	1.80832	1.80832	1.45268
$v_{10}$	1.27021	1.80832	1.63661	1.54063	1.27021	1.45260
$v_{11}$	4.60679	−2.72137	10.20329	10.15968	4.60679	2.24190
$v_{12}$	7.38990	10.84531	−0.75565	−0.07147	7.38990	8.58425
$v_{13}$	1.62547	2.18505	−0.19032	−0.01800	1.62547	1.81998
$i_{V_{S1}}$	−0.00685	−0.00787	−0.00725	−0.00729	−0.00685	−0.00652
$i_{V_{S2}}$	−0.00034	−0.00021	−0.00047	−0.00044	−0.00034	−0.00038
$i_{V_{S3}}$	0.00002	0.00007	0.00002	−0.00001	0.00002	0.00016
$\hbox {Error}_{15}$	3.7168E−11	5.4131E−9	4.8390E−12	4.4469E−5	1.6678E−9	2.5092E−6
$\hbox {Error}_{7}$	1.1229E−7	1.4545E−7	1.2585E−7	4.4469E−5	1.1240E−7	2.5007E−6

### Example 3

**Fig. 15:**
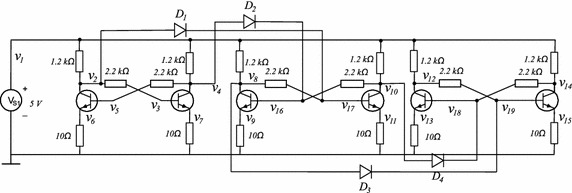
Proposed circuit for Example 3

This circuit has been proposed for this work (see Fig. 15). Contains three flip-flops connected in a cascade configuration. The transistors are modelled using the Ebers–Moll model. The V–I characteristics for diodes $\hbox {D}_1, \hbox {D}_2, \hbox {D}_3$, and $\hbox {D}_4$ are given by

17 $$\begin{aligned} i_{D}&=-66.24887+78.72290v_{D}+0.28774E-2|v_{D}|+.9397385778|v_{D}-0.7| \\&\quad +10.44408|v_{D}-0.8|+67.33621|v_{D}-.85|, \end{aligned}$$

where $v_D$ represents the voltage drop in diode *D* and $i_D$ represents its current.

**Fig. 16:**
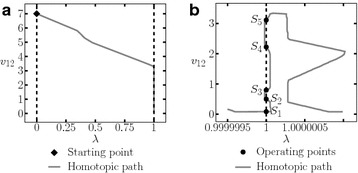
Homotopic path for $v_{12}$ from the Example 3. **a** Homotopic path for $v_{12}$. **b** Zoom to **a**

This circuit contains twenty variables; calculating one SP (listed in Table 7) found five different operating points. Table 8 shows the operating points and Fig. 16 presents the homotopy path for $v_{12}$.

**Table 7:** Starting point selected for Example 3

Variable	SP
$v_{1}$	10
$v_{2}$	−5
$v_{3}$	0
$v_{4}$	8
$v_{5}$	0
$v_{6}$	−8
$v_{7}$	−6
$v_{8}$	10
$v_{9}$	7
$v_{10}$	−2
$v_{11}$	−1
$v_{12}$	7
$v_{13}$	9
$v_{14}$	−8
$v_{15}$	−1
$v_{16}$	0
$v_{17}$	0
$v_{18}$	0
$v_{19}$	0
$i_{V_{S1}}$	−1

**Table 8:** Operating points for Example 3

Solution	$S_1$	$S_2$	$S_3$	$S_4$	$S_5$
Variable					
$v_{1}$	5	5	5	5	5
$v_{2}$	0.68446	0.78476	0.78492	0.78494	1.00839
$v_{3}$	0.64634	0.71514	0.71519	0.71520	0.71942
$v_{4}$	1.00839	0.78494	0.78492	0.78476	0.68447
$v_{5}$	0.71942	0.71520	0.71519	0.71514	0.64635
$v_{6}$	0.03487	0.03175	0.03174	0.03169	0.01737
$v_{7}$	0.01736	0.03169	0.03174	0.03175	0.03488
$v_{8}$	0.19391	0.63029	0.63306	0.63369	1.02235
$v_{9}$	0.05758	0.03918	0.03978	0.03975	0.01735
$v_{10}$	1.02234	0.63369	0.63306	0.63029	0.19391
$v_{11}$	0.17351	0.03975	0.03978	0.03918	0.05758
$v_{12}$	0.08463	0.50526	0.79630	2.21665	3.12335
$v_{13}$	0.06687	0.04471	0.03503	0.01662	0.00497
$v_{14}$	3.12334	2.21665	0.79630	0.50526	0.08464
$v_{15}$	0.00497	0.01662	0.03503	0.04471	0.06687
$v_{16}$	0.75199	0.72532	0.72606	0.72601	0.64577
$v_{17}$	0.64577	0.72601	0.72606	0.72532	0.75200
$v_{18}$	0.76623	0.73329	0.71937	0.61847	0.18512
$v_{19}$	0.18511	0.61847	0.71937	0.73329	0.76624
$i_{V_{S1}}$	−0.01990	−0.02037	−0.02130	−0.02037	−0.01990
$\hbox {Error}_{15}$	4.7344E−9	9.1796E−8	6.1874E−9	2.5534E−8	4.6363E−9
$\hbox {Error}_{7}$	2.7554E−8	9.1849E−8	3.9932E−8	3.6188E−8	2.1458E−8

### Example 4

**Fig. 17:**
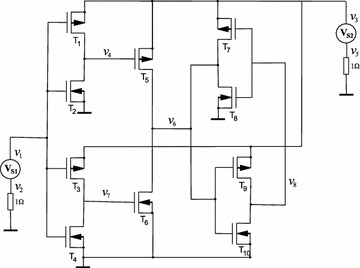
Schematic circuit for Example 4

**Fig. 18:**
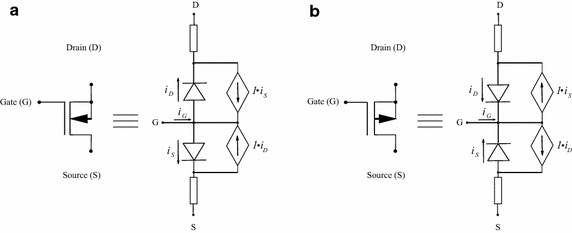
Shichmann–Hodges model for CMOS transistors. **a** Model for NMOS transistors. **b** Model for PMOS transistors

This circuit (see Fig. 17) was studied in Tadeusiewicz and Kuczynski (2013); it contains five NMOS transistors and five PMOS transistors. The CMOS transistors are represented by using the Shichmann–Hodges model (Shichman and Hodges 1968) (Fig. 18) and simulated in SPICE setting the following parameters: LEVEL $=$ 1, VT0 $=$ 0.5705, RD $=$ RS $=$ 0, LAMBDA $=$ 0. For NMOS transistors K$_p$ $=$ 79.173u, W $=$ 51u, L $=$ 4u, and for PMOS K$_p$ $=$ 19.485u, W $=$ 102u, L $=$ 2u. The V–I characteristics for NMOS and PMOS transistors are given by Adby (1980), Tadeusiewicz (2001) and Tadeusiewicz and Kuczynski (2013)

18 $$\begin{aligned} i_S(v_G,v_S)&= {\left\{ \begin{array}{ll} 0 &{} \quad \text {if }v_G-v_S< 0, \\ k(v_G-v_S-v_{th})^2 &{} \quad \text {if }v_G-v_S \ge 0, \\ \end{array}\right. } \\ i_D(v_G,v_D)&= {\left\{ \begin{array}{ll} 0 &{} \quad \text {if }v_G-v_D < 0, \\ k(v_G-v_D-v_{th})^2 &{} \quad \text {if }v_G-v_D \ge 0, \\ \end{array}\right. } \end{aligned}$$

where $v_G$ represents the voltage at the gate node, $v_S$ the voltage at the source node, $v_D$ the voltage at the drain node, $i_S$ and $i_D$ represent the branch currents in source and drain, respectively.

The value of *k* is calculated (Tadeusiewicz and Kuczynski 2013) as

19 $$\begin{aligned} k= \frac{K_pW}{2L}. \end{aligned}$$

Using (19) and (18) to set the PWL models; NMOS transistors are modelled as

20 $$\begin{aligned} i_{S}(v_g,v_S)&=.21475k|v_{G}-v_{S}-.57050|+.71475k|v_{G}-v_{S}-1| \\&\quad +k|v_{G}-v_{S}-2|+1.5k|v_{G}-v_{S}-3|+2k|v_{G} \\&\quad -v_{S}-5|+5.42950k v_{G}-v_{S}-17.33726k, \\ i_{D}(v_g,v_D)&=.21475k|v_{G}-v_{D}-.57050|+.71475k|v_{G}-v_{D}-1| \\&\quad +k|v_{G}-v_{D}-2|+1.5k|v_{G}-v_{D}-3|+2k|v_{G} \\&\quad -v_{D}-5+5.42950k v_{G}-v_{D}-17.33726k, \end{aligned}$$

as for the PMOS transistors, the model is as follows

21 $$\begin{aligned} i_{S}&= 0.08245k|v_{G}-v_{S}|-.835101+.58245k|v_{G}-v_{S}-1| \\&\quad +k|v_{G}-v_{S}-2|+1.5k|v_{G}-v_{S}-3|+2k|v_{G}-v_{S}-5| \\&\quad +5.16490k v_{G}-v_{S}-17.15130k, \\ i_{D}&= 0.08245k|v_{G}-v_{D}|-.835101+.58245k|v_{G}-v_{D}-1| \\&\quad +k|v_{G}-v_{D}-2|+1.5k|v_{G}-v_{D}-3|+2k|v_{G}-v_{D}-5| \\&\quad +5.16490k v_{G}-v_{D}-17.15130k. \end{aligned}$$

**Fig. 19:**
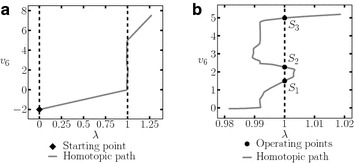
Homotopic path from Example 4. **a** Homotopic path of $v_6$. **b** Zoom to **a**

Using the SP shown in Table 9, three operating points were found (Table 10). Figure 19a shows the resulting homotopic path for $v_6$ and Fig. 19b a zoom to the homotopic path, it is possible to notice the location of the operating points more clearly.

**Table 9:** Starting point for Example 4

Variable	SP1
$v_{1}$	−2
$v_{2}$	−2
$v_{3}$	−2
$v_{4}$	12
$v_{5}$	−2
$v_{6}$	−2
$v_{7}$	12
$v_{8}$	12
$i_{V_{S2}}$	−2
$i_{V_{S1}}$	−2

**Table 10:** Operating points found for Example 4

Solution	$S_1$	$S_2$	$S_3$
Variable			
$v_{1}$	2.5	2.5	2.5
$v_{2}$	−4.91517E−12	−2.76401E−12	1.10556E−12
$v_{3}$	4.99124	4.99026	4.99694
$v_{4}$	1.06307	1.06132	1.07323
$v_{5}$	−0.00875	−0.00973	−0.00030
$v_{6}$	1.49486	2.26321	4.98690
$v_{7}$	1.06307	1.06132	1.07323
$v_{8}$	4.82941	3.80844	0.00146
$i_{V_{S1}}$	−4.91517E−12	−2.76401E−12	−1.10556E−12
$i_{V_{S2}}$	−0.00875	−0.00973	−0.00305
$\hbox {Error}_{15}$	1.2340E−11	1.0773E−11	5.0228E−6
$\hbox {Error}_{7}$	1.0201E−7	4.9503E−7	5.0260E−6

### Example 5

**Fig. 20:**
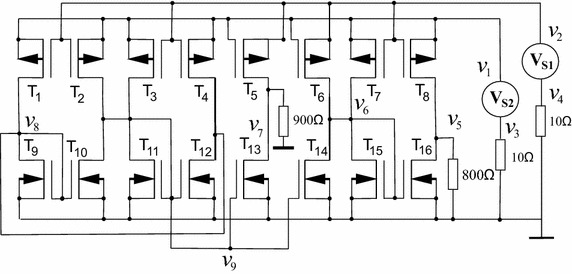
Schematic circuit for Example 5

The circuit shown in Fig. 20 was introduced in Tadeusiewicz and Kuczynski (2013), it has three operating points. The transistors in this circuit were modelled using (20) and (21) and setting $k=0.5$ mA/V^2^ for every transistor, except for T12 and T4, for both transistors it was set to $k=1$ mA/V^2^. The model was simulated in SPICE using the parameter values given in Example 4, except for T12 and T4, their parameters were set as follows: W = 51u, L = 2u, and W = 102u, L = 1u, respectively.

**Fig. 21:**
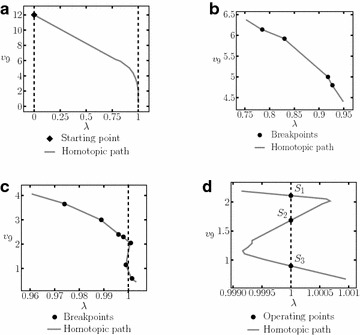
Homotopic path for $v_2$ from Example 5. **a** Homotopic path for $v_2$. **b** Zoom to **a**. **c** Zoom to **a**. **d** Zoom to **a**

The SP for the circuit is shown in Table 11. It was possible to find three solutions (see Table 12). The homotpic path is shown in Fig. 21. The obtained solutions are shown in Fig. 21d.

**Table 11:** Starting point for Example 5

Variable	SP
$v_{1}$	6
$v_{2}$	−9
$v_{3}$	6
$v_{4}$	6
$v_{5}$	12
$v_{6}$	−9
$v_{7}$	−9
$v_{8}$	−9
$v_{9}$	12
$i_{V_{S2}}$	6
$i_{V_{S1}}$	6

**Table 12:** Operating points found for Example 5

Solution	$S_1$	$S_2$	$S_3$
Variable			
$v_{1}$	4.94280	4.94280	4.94280
$v_{2}$	3	2.99999	3
$v_{3}$	−0.05719	−0.05719	−0.05719
$v_{4}$	−4.9428E−12	−5.9576E−12	−1.1418E−12
$v_{5}$	0.39613	0.29148	0.24268
$v_{6}$	1.07929	1.49869	2.10639
$v_{7}$	0.25796	0.31132	0.51159
$v_{8}$	0.87633	1.50263	2.37228
$v_{9}$	2.10891	1.68416	0.89835
$i_{V1}$	−4.5210E−12	−5.9576E−12	−4.5210E−12
$i_{V2}$	−0.00571	−0.00571	−0.00571
$\hbox {Error}_{15}$	1.3441E−11	9.4875E−11	2.3965E−6
$\hbox {Error}_{7}$	1.8000E−7	1.8001E−7	1.8000E−6

## Numerical simulation and discussion

This section presents a performance comparison between the SHPT method proposed in this work and the MSA proposed in Vazquez-Leal et al. (2014). Also, the MSA method from Vazquez-Leal et al. (2014) was used without any modification. Besides, the inverted sphere technique was also applied to the MSA method and its performance evaluated. As will be seen, the SHPT path tracking method reduced, significantly, the computing time and the inverted sphere technique allowed, efficiently, to avoid the reversion phenomenon. Besides, an starting point criteria was employed that, in fact, eased the process to find multiple operating points using SHPT and MSA.

Tables 13, 14, and 15 show the comparison between the performance of three different simulations for each example studied in the previous section. SHPT stands for the proposed path tracking method, MSA2 is the method introduced in Vazquez-Leal et al. (2014), MSA1 is the method from Vazquez-Leal et al. (2014) but including the inverted hypersphere technique which is a proposal of this work.

**Table 13:** Comparison between the SHPT and the MSA path tracking methods

Example	1			2			2		
Starting point	SP1			SP1			SP2		
Characteristic	SHPT	MSA1	MSA2	SHPT	MSA1	MSA2	SHPT	MSA1	MSA2
Total iterations	360	360	360	2500	2500	2500	1400	1400	2
Hypersphere radius	0.001	0.001	0.001	0.05	0.05	0.05	0.05	0.05	0.05
Reversion phenomenon	No	No	No	No	No	No	No	No	Yes
Solutions found	3	3	3	4	4	4	2	2	0
Straight Line iterations	336	0	0	2443	0	0	1376	0	0
Hypersphere iterations (NRM)	24	360	360	57	2500	2500	24	1400	2
Total computing time (s)	4.30	22.33	22.27	10.71	92.44	92.36	7.05	56.08	*
Straight Line computing time	0.359	0	0	3.04	0	0	1.06	0	*
Hyperspheres computing time	3.94	22.33	22.27	7.67	92.44	92.36	5.99	56.08	*

**Table 14:** Comparison between the SHPT and the MSA path tracking methods

Example	2			3			4		
Starting point	SP3			SP1			SP1		
Characteristic	SHPT	MSA1	MSA2	SHPT	MSA1	MSA2	SHPT	MSA1	MSA2
Total iterations	2000	2000	1501	1000	1000	345	400	400	2
Hypersphere radius	0.05	0.05	0.05	0.1	0.1	0.1	0.1	0.1	0.1
Reversion phenomenon	No	No	Yes	No	No	Yes	No	No	Yes
Solutions found	3	3	2	5	5	0	3	3	0
Straight Line iterations	1937	0	0	802	0	0	214	0	0
Hypersphere iterations (NRM)	63	2000	1501	198	2000	345	186	400	2
Total computing time (s)	12.48	87.65	*	224.73	798.16	*	48.75	90.35	*
Straight Line computing time	1.41	87.65	*	6.56	0	*	1.14	0	*
Hyperspheres computing time	11.07	87.65	*	218.16	798.16	*	47.61	90.35	*

**Table 15:** Comparison between the SHPT and the MSA path tracking methods

Example	5		
Starting point	SP1		
Characteristic	SHPT	MSA1	MSA2
Total iterations	330	330	2
Hypersphere radius	0.1	0.1	0.1
Reversion phenomenon	No	No	Yes
Solutions found	3	3	0
Straight Line iterations	249	0	0
Hypersphere iterations (NRM)	81	330	2
Total computing time (s)	26.36	61.49	*
Straight Line computing time	1.09	0	*
Hyperspheres computing time	25.27	61.49	*

Example 1 and example 2 did not show any sign of the reversion phenomenon in any of the simulations performed. Nevertheless, the rest of the simulations using the MSA2 method showed reversion. No simulation using the MSA1 method showed reversion. It is important to make notice of the fact that the homotopic path is the same for the three types of simulation; this is because they are based on the same homotopy formulation, and the difference lies in the applied path tracking method. The results prove that the efficiency is improved using the inverted sphere technique proposed in this work, as it helped to avoid the revision phenomenon and allowed to perform the path tracking without issues. The cases where reversion phenomenon were noticed are marked with an asterisk (*), the computing time for these cases was not possible to be calculated because the method locked and no further calculations were possible.

As shown in Tables 13, 14, and 15, the time spent in straight line iterations is minimum compared to the time spent in iterations using hyperspheres. This characteristic allows the acceleration of the path tracking.

Computing time increases as the density of breakpoints grows. Nevertheless, for the worst case scenario, a curve with high density of breakpoints or a non-PWL curve, the proposed method would only spend the same computing time as the method proposed in Vazquez-Leal et al. (2014). The computing time spent by the MSA1 simulations and the SHPT method is noticeable different; the SHPT method performed up to twelve times faster. For the worst case scenario, the difference was 1.89 times faster than MSA1. The algorithm was implemented in Maple 15. Future work will focus on implementing the technique in Fortran, the goal is to improve the computation time and analyse larger circuits.

## Conclusions

This work introduced a path algorithm for analysis of PWL circuits using the HCM, this algorithm exhibited improvements in the computing time compared to the algorithm proposed in Vazquez-Leal et al. (2014). Also, a starting point criteria was proposed in order to achieve better performance of the HCM, this criterion was proved useful but it does not assure an homotopic path that travels through all the root of the system. It just increase the probability of finding them. Furthermore, a technique for avoiding the reversion method was suggested and proved to be effective. By experimentation was possible to avoid reversion and allowed the continuation of the path tracking, nevertheless, for systems with a high density of variables (around one hundred) some instabilities were detected. Further work on this technique aims to improve the number of variables that is possible to work on. As it can be seen in Tables 13, 14, and 15, most of the simulation time is spent in NRM iterations, this leads to focus future work on the reduction of time spent on them. As a final comment, the SHPT path tracking method does not have a stop criterion, thus, the development of a stop criterion would be an important improvement.
